# The *beta-1*, *4-N-acetylglucosaminidase 1* gene, selected by domestication and breeding, is involved in cocoon construction of *Bombyx mori*

**DOI:** 10.1371/journal.pgen.1008907

**Published:** 2020-07-15

**Authors:** Chunlin Li, Xiaoling Tong, Weidong Zuo, Hai Hu, Gao Xiong, Minjin Han, Rui Gao, Yue Luan, Kunpeng Lu, Tingting Gai, Zhonghuai Xiang, Cheng Lu, Fangyin Dai

**Affiliations:** State Key Laboratory of Silkworm Genome Biology, Key Laboratory of Sericultural Biology and Genetic Breeding, Ministry of Agriculture and Rural Affairs, College of Biotechnology, Southwest University, Chongqing, China; South China Normal University, CHINA

## Abstract

Holometabolous insects have distinct larval, pupal, and adult stages. The pupal stage is typically immobile and can be subject to predation, but cocoon offers pupal protection for many insect species. The cocoon provides a space in which the pupa to adult metamorphosis occurs. It also protects the pupa from weather, predators and parasitoids. Silk protein is a precursor of the silk used in cocoon construction. We used the silkworm as a model species to identify genes affecting silk protein synthesis and cocoon construction. We used quantitative genetic analysis to demonstrate that *β-1*,*4-N-acetylglucosaminidase 1* (*BmGlcNase1*) is associated with synthesis of sericin, the main composite of cocoon. *BmGlcNase1* has an expression pattern coupled with silk gland development and cocoon shell weight (CSW) variation, and CSW is an index of the ability to synthesize silk protein. Up-regulated expression of *BmGlcNase1* increased sericin content by 13.9% and 22.5% while down-regulation reduced sericin content by 41.2% and 27.3% in the cocoons of females and males, respectively. Genomic sequencing revealed that sequence variation upstream of the *BmGlcNase1* transcriptional start site (TSS) is associated with the expression of *BmGlcNase1* and CSW. Selective pressure analysis showed that *GlcNase1* was differentially selected in insects with and without cocoons (*ω*_*1*_ = 0.044 vs. *ω*_*2*_ = 0.154). This indicates that this gene has a conserved function in the cocooning process of insects. *BmGlcNase1* appears to be involved in sericin synthesis and silkworm cocooning.

## Author summary

The cocoon provides a protected space for the metamorphosis of many insect species. Silk protein is a precursor of the fiber used for cocoon construction. Deciphering the genetic basis underlying silk protein synthesis will improve our understanding of cocoon construction and the adaptations of species that construct cocoons. We used the silkworm (*Bombyx mori*) as a model to identify genes affecting silk protein synthesis and cocoon construction. Quantitative genetic analysis was used to show that *β-1*,*4-N-acetylglucosaminidase 1* (*BmGlcNase1*), a gene selected during silkworm domestication and breeding, is associated with sericin synthesis. Transgenic-based functional validation confirmed that *BmGlcNase1* positively regulates sericin content in the silkworm cocoon. The selective pressure of *GlcNase1* in the evolution of insects with cocoons is higher than those without cocoons. This indicates that it has a conserved function in the cocooning process. These results reveal aspects of the genetic basis of silk protein synthesis and the cocoon construction of insects.

## Introduction

Insects have evolved adaptations for each developmental stage and this has helped them become a successful group. Cocoons are an adaptation that protects insect pupae. For example, the wild silkworm (*Bombyx mandarina*) camouflages its pupa by using its silk to wrap mulberry leaves around the cocoon. *Antherea pernyi* and *Antheraea yamamai* spin cocoons with natural colors adapted to their environments for concealment. Cocoon adaptions help protect insects from predators and parasites. At least 17 insect orders have species with the ability to spin silk. Many insects construct cocoons during a specific developmental stage and this is usually the final instar [1]. Deciphering the genetic and evolutionary basis underlying cocoon construction will advance our understanding of the adaptations of these insects.

The silkworm (*Bombyx mori*) is the best example of an insect that spins cocoons. The silkworm cocoon is a capsule structure enclosed by silk. It provides a strong, breathable space that is waterproof. An intact cocoon is spun with a single continuous silk fiber that ranges between 500 m to 1500 m in length [2]. This silk fiber is mainly composed of fibroin and sericin proteins. Fibroin serves as the scaffold of the cocoon, while sericin acts as an adhesive to bind the silk fibroin. Cocoon spinning is a complex physiological behavior, involving synthesis, secretion, assembly, and spinning of the silk proteins. Silk research has mainly focused on silk assembly and spinning behavior [3, 4]. It is noteworthy that silk proteins, including sericin and fibroin, are the material basis of cocoons. An appropriate amount of silk protein is needed for cocoon construction. Studying the molecular basis of silk protein synthesis provides insight into the genetic and evolutionary basis of cocoon construction.

The silkworm is the only domesticated invertebrate producing economic quantities of silk. The domestication process began > 7500 years ago [5, 6]. Long-term domestication and breeding greatly improved the silk protein synthesis of the domestic silkworm compared to its wild counterpart (*Bombyx mandarina)*. The cocoon shell weight (CSW), an important index for silk protein content, increased from 0.05 g to 0.5 g. Domestication and breeding also produced several silkworm strains with varying ability to synthesize silk protein. These strains can be used to study the genetic basis underlying the synthesis of silk protein. We used the silkworm as a model to study the genetic basis underlying silk protein synthesis and cocoon construction.

The ability to synthesize silk protein, measured by CSW, is a quantitative trait [2]. Several studies have explored the genetic basis of this trait using quantitative trait loci (QTL) mapping [7–11] and selection analysis [12, 13]. However, fine mapping and cloning of genes that affect silk protein synthesis has not been done. We previously identified a major locus on the 11^th^ chromosome that controls CSW [11, 14]. In this study, we performed a genetic analysis based on selective genotyping and association analysis. We identified a gene (*BmGlcNase1*) that affects the synthesis of sericin and is involved in the formation of silkworm cocoons.

## Results

### Genetic analysis associates *BmGlcNase1* with variation in CSW

**Fig 1:**
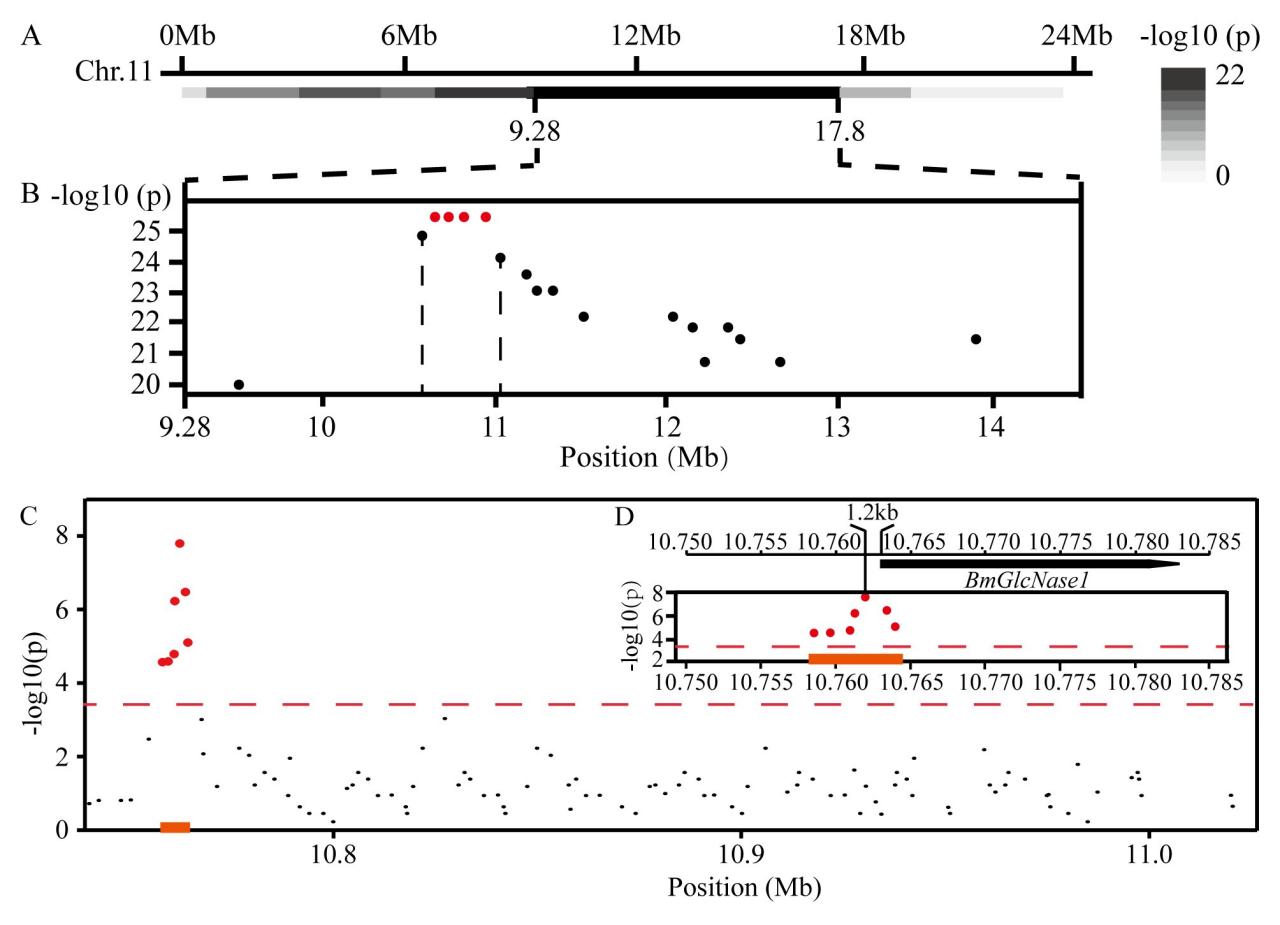
Genetic analysis of *csw2*. (A) Preliminary mapping based on selective genotyping. The genomic position of *csw2* was first confirmed by genotyping the markers used in preliminary QTL mapping (Part 1 in S1 Table) [11]. The linkage significance (p) between each marker and the CSW was calculated by one-way ANOVA. The line with the ruler above represents the 11^th^ chromosome; the grey color bar shows the negative logarithm of linkage significance of the corresponding chromosome section. Chr, chromosome; Mb, mega base pair. (B) Fine mapping of *csw2* by selective genotyping. Each point in the frame represents an Indel marker (Part 2 in S1 Table) developed in *csw2* and red points are the Indels with the highest linkage significance. The linkage significance (p) between each Indel and CSW was calculated by one-way ANOVA. (C) Association analysis based on germlines. One-way ANOVA was used to calculate the association significance between each Indel and CSW. The Bonferroni adjustment was used to obtain the threshold in association analysis. Each point represents an Indel marker and red points are the Indels with significance lower than the threshold (associated Indel). Numbers below indicate the genomic location of Indels. (D) Diagram of the association region with red highlights. Line with the ruler above represents the genomic region containing *BmGlcNase1*. The black frame represents the *BmGlcNase1* gene and the arrow shows the gene direction. The solid red line shows the genomic location of the associated Indels.

Preliminary QTL analysis was made using IS-Dazao and 872B, a pair of silkworm strains with varied CSW, as parents. We mapped csw2, a locus that explained 15.07% of the variation of CSW in a segregating population, to the region bracketed by markers at 9.2 and 17.8 mega base pair (Mb) on the 11^th^ chromosome (Fig 1A) [11, 14]. To precisely identify the mapping region, we performed a selective genotyping experiment. IS-Dazao and 872B were also used as parents. Two subgroups included 200 males with low CSW values and the another 200 males with high CSW values were selected from a backcross population containing 4325 individuals [11, 15]. Via chromosome walking, we identified four sequential indels with the highest linkage significance in the region between 10.74 and 11.01 Mb (Fig 1B). The genotyping result of these indels in the 400 individuals suggests that they were tightly linked.

We conducted an association analysis using the germplasm strains conserved in the silkworm gene bank. Ninety-nine Indel markers were developed in this region and 95 germlines with normal viability and distant kinship were selected as the association analysis population. Selection was based on germplasm conservation and breeding experience (S8 Table). This population exhibited substantial CSW variation. The XiaFang strain had the highest CSW (0.383 g) while strain 19–460 had the lowest CSW (0.078 g). By genotyping the indels in the association analysis population, we identified a cluster of indels associated with CSW (Fig 1C). Among these, Indel-10.762 had the highest association significance. This indel is located 1.2 kilo base pairs (kb) upstream of the transcription start site (TSS) of gene *BMgn011646*. This suggests that *BMgn011646* may be involved in the control of CSW (Fig 1D). Functional annotation showed that *BMgn011646* encodes *β-1*,*4-N-acetylglucosaminidase 1* (*BmGlcNase1*).

### Expression of *BmGlcNase1* is correlated with silk gland development and CSW

**Fig 2:**
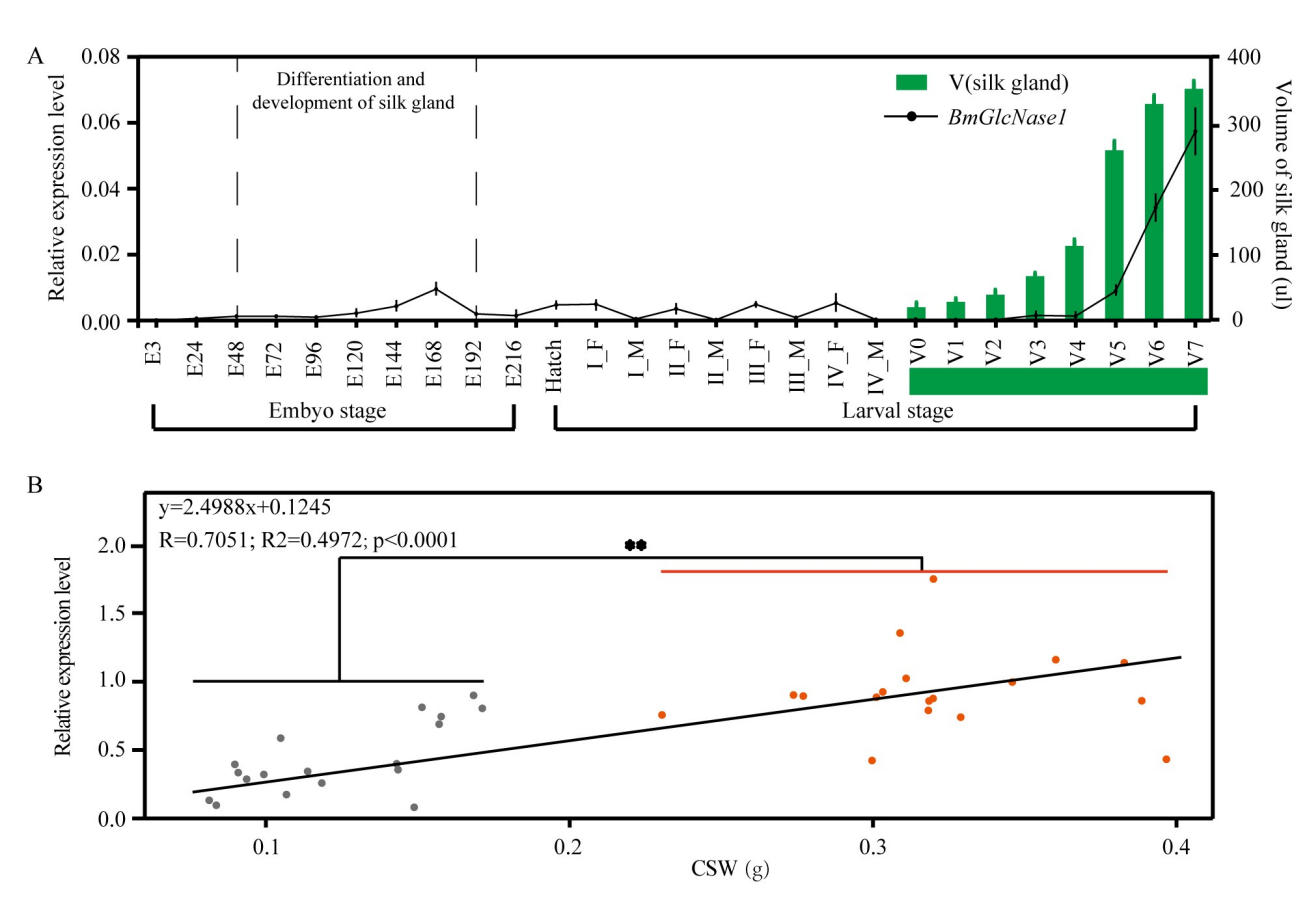
Expression pattern of *BmGlcNase1*. (A) Correlation between silk gland development and temporal expression of *BmGlcNase1*. Dot plot shows the relative expression level of *BmGlcNase1* in each sample. E3-E216, the embryo at 3–216 hours after incubation; Hatch, the new hatched larvae; I_F, feeding phase in the 1^st^ instar; I_M, molting phase in the 1^st^ instar; I-V, the 1^st^ to the 5^th^ instar; V0-V7, start to the 7^th^ day of the 5^th^ instar; the height of the green bar represent the volume of silk gland. Each point represents the average of 3 replicate and the error bar shows the standard deviation (SD.). (B) Association analysis between the expression level of *BmGlcNase1* and CSW of varied silkworm strains. Each point represents the average expression level of *BmGlcNase1* in the silk gland of one silkworm strain. The grey points represent strains with relatively lower CSW, while the red points represent these with higher CSW. Three replicates were performed in this experiment with silk glands from three larvae in each replicate. Linear regression analysis was used to determine the correlation between the expression level of *BmGlcNase1* and CSW. Two tailed Student’s *t*-test was used to compare the expression difference between the silkworm with lower and higher CSW. ** represents a significant difference at the 0.01 level.

The associated Indel is located upstream of the TSS of *BmGlcNase1*, indicating that the transcription level of this gene may be associated with CSW. Therefore, we investigated the expression pattern of *BmGlcNase1*. The spatial expression pattern shows that it has a relatively specific high expression in the middle silk gland (S1A Fig), but no expression was detected in the posterior silk gland. Silk glands are silkworm tissues that synthesize silk protein. They consist of anterior, middle, and posterior parts. The middle and posterior silk glands synthesize sericin and fibroin proteins, respectively. The volume study of parental silk glands showed that 872B, the parent with higher CSW, had a larger silk gland compared to IS-Dazao, the parent with lower CSW (S1B Fig). We determined the temporal expression profile of *BmGlcNase1*. *BmGlcNase1 was* mainly expressed in the middle and late stages of embryonic development and in the fifth instar which are key stages for silk gland development (Fig 2A). Silk gland cell division and active endomitosis during these stages determines the number and size of silk gland cells and thus the final silk gland volume. The expression pattern of *BmGlcNase1* was highly associated with silk gland development. In the fifth instar, the daily transcription level was highly associated with the silk gland volume (Fig 2A). These results indicate that *BmGlcNase1* is involved in silk gland development.

We surveyed the expression of this gene in the middle silk gland of additional strains with varied CSW. Thirty-six strains were allowed to feed up to the last day of the fifth instar (S8 Table). The middle silk gland of each was then dissected at this time because the gene exhibited high expression on this day. Transcript level analysis showed a significant association between gene expression and CSW (r = 0.7051) (Fig 2B; *p*<0.0001). Based on the CSW performance of each strain, we divided the strains into two groups with low and high CSW. We then compared the expression level of each group. There was significantly higher expression in the group with higher CSW than in the low CSW group (Fig 2B). This suggests that *BmGlcNase1* may play an important role in silk gland development and may be associated with the CSW of silkworms.

### A cluster of noncoding variants was associated with *BmGlcNase1* expression and CSW

**Fig 3:**
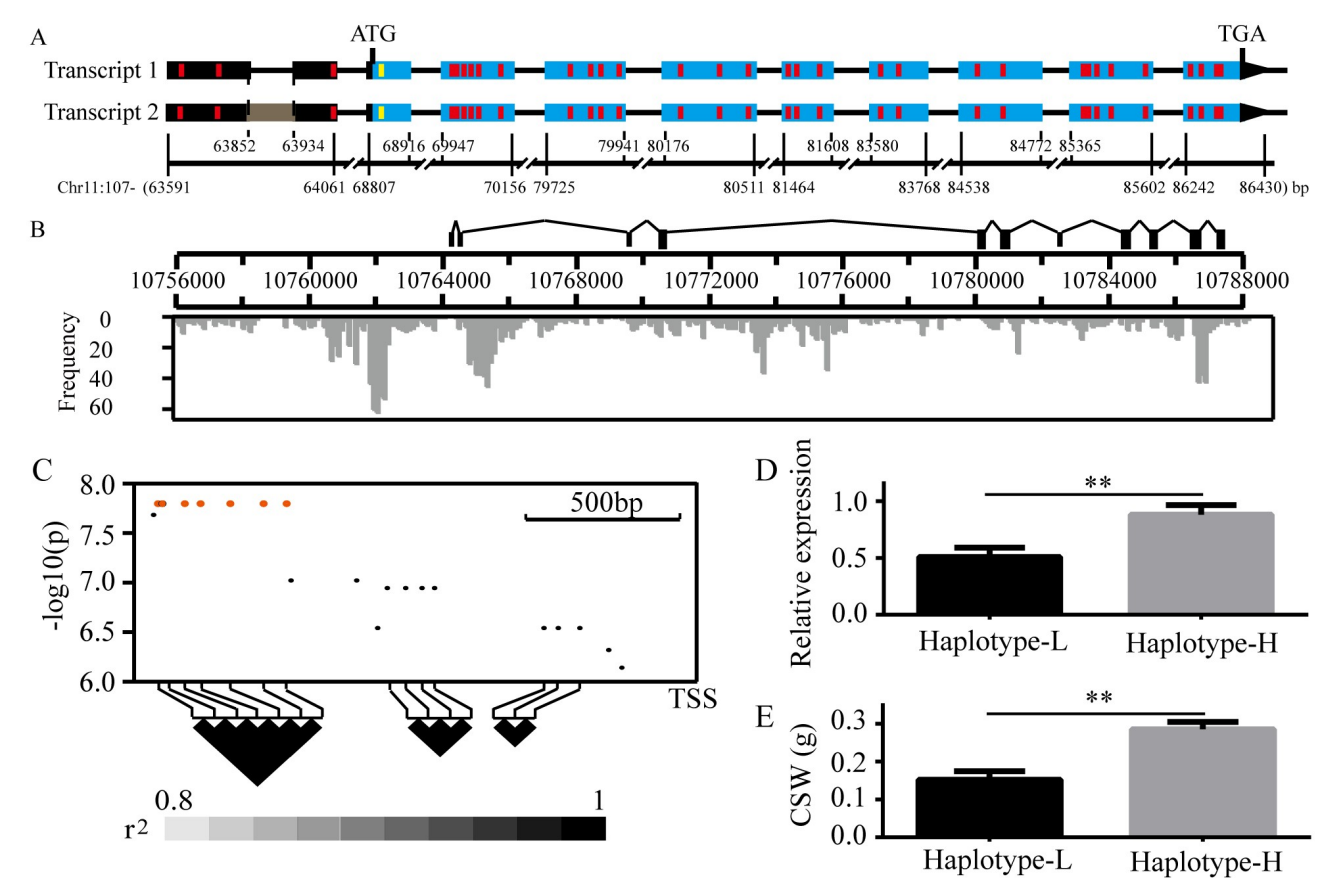
Sequence variation of *BmGlcNase1*. (A) cDNA sequence and gene structure of *BmGlcNase1*. The wireframes represent the structure of the two *BmGlcNase1* transcripts, of which the blue frames indicate the exons in open reading frame (ORF) and the blacks indicate the untranslated exons. The horizontal lines indicate the introns. The grey frame in transcript 2 shows the inserted fragment in this transcript. Red vertical bars indicate the synonymous SNPs and the yellow bar shows the synonymous SNP. The ruler below shows the position of each part of *BmGlcNase1* on the 11^th^ chromosome. The 107- (63591–86430) indicate the 10,763,591 to 10,786,430 nt of the 11^th^ chromosome. (B) Genomic sequence comparison of *BmGlcNase1* between parents; columns represent the number of variants in this region. The combination of wireframes and ruler showed the gene structure of *BmGlcNase1*. The grey histogram shows the number of SNP/Indels in each 100 bp bin along the genomic region covering *BmGlcNase1*. (C) Association analysis between upstream indels and CSW. Each point indicates the association significance of one Indel upstream of *BmGlcNase1*, of which the red points show those with the highest significance. The paired linkage disequilibrium (r^2^) values are shown at the bottom. (D-E) Comparison of *BmGlcNase1* expression and CSW between strains with L and H haplotypes. Two tailed Student’s *t*-test was used to compare the two indexes between the two groups. Error bar, SD. ** represents a significant difference at the 0.01 level.

We used rapid amplification of cDNA ends (RACE) to obtain the full cDNA of parents, IS-Dazao and 872B, and analyzed their gene structure. *BmGlcNase1* contains 11 exons; the open reading frame (ORF) starts at the 3^rd^ exon and ends at the 11^th^ exon (Fig 3A). Two types of transcript were detected in both strains, IS-Dazao and 872B. Compared with the type I transcript, there was one 106 bp fragment insertion in the second exon of the type II transcript. However, there was no variation in the ORF region between them (Fig 3A). The sequence of the type I transcript in Is-Dazao and 872B was then compared. There were 33 SNPs in the full cDNA and, among them, three were located in the 5’ untranslated region (UTR), and another 30 SNPs were in the ORF (Fig 3A). Amino acid (AA) analysis showed that only the first SNP in the ORF led to the substitution (Gly-Arg). Protein structure analysis suggested that this substitution was in the signal peptide, but no signal peptide characteristic difference was detected between the coding products of IS-Dazao and 872B. These results indicated that variation in the *BmGlcNase1* transcription level might be associated with the variation in CSW.

We determined the genomic sequence of both parents. In total, 30 kb of the genomic region covering *BmGlcNase1* was sequenced. A large number of variations were detected in this region (2,242), including 1932 SNPs and 309 indels. These variations were mainly enriched in the upstream and intron regions, rather than distributed evenly (Fig 3B). The association analysis showed that indels in the upstream region were associated with CSW. We thus genotyped indels in the 1.5 kb upstream region of TSS in the association analysis population and calculated their association significance with CSW. Indels in the region between 1631 bp and 1264 bp exhibited the highest association with CSW (p = 1.59e-08) (Fig 3C) (S5 Table). Genotyping results of multiple strains showed that these associated indels were grouped into two haplotypes (L/H). CSW significantly varied between the two haplotypes (Fig 3D). The *BmGlcNase1* gene exhibited significantly higher expression in H haplotype strains than that in L haplotypes (Fig 3E). These results suggest that variants in this region may contribute to the expression variation of *BmGlcNase1* and the CSW differences among silkworm strains.

### Functional validation showed *BmGlcNase1* has a significant effect on sericin synthesis

**Fig 4:**
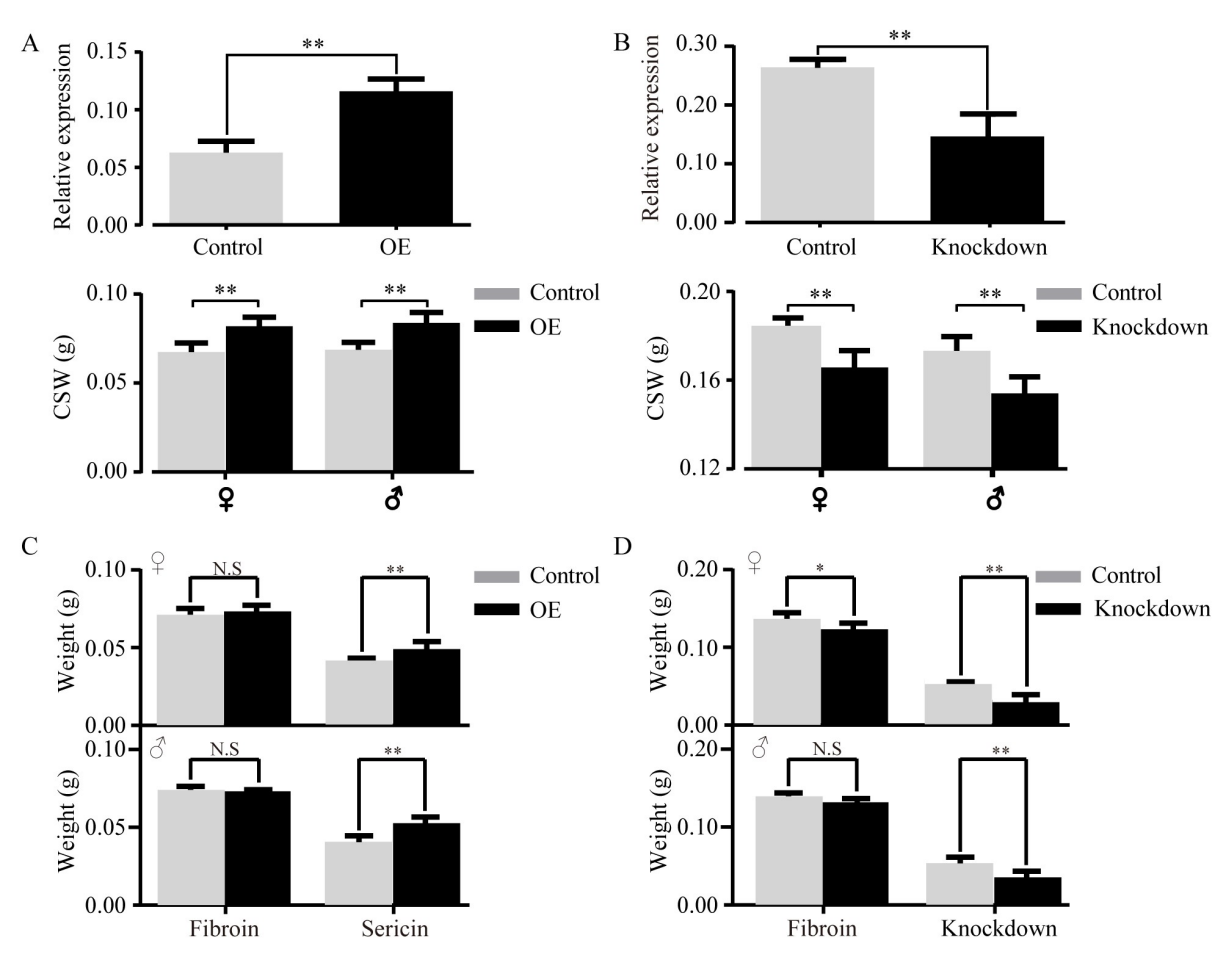
Functional analysis of *BmGlcNase1*. (A-B) Overexpression and knockdown of *BmGlcNase1* and the effect on CSW. The above histograms in both (A) and (B) show the expression level of *BmGlcNase1* in control and over-expression groups and the lows show the CSW of the two groups. OE, over-expression. For expression level determination, three replicates were performed with silk glands from three larvae in each sample. (C-D) Effect of *BmGlcNase1* overexpression and knockdown on the synthesis of sericin and fibroin. Two tailed Student’s *t*-test was used for comparison. Error bar, SD. N.S., no significant; * and ** represented significant differences at the 0.01 level.

To study the effect of *BmGlcNase1* on CSW, we used transgenic technology to elevate and knockdown its expression in the middle silk gland (S2 Fig) by transgenic technology. Reverse PCR indicated that the insertion site of the overexpression and interference constructs is located in the intergenic region, which rules out the disruption of other silkworm genes (S2B and S2E Fig). To avoid interference with the feeding environment, 150 normal and 150 transgenic newly hatched larvae were mixed for feeding until the wandering stage, during which the larvae search for places to spin their cocoons. During the wandering stage, the larvae were differentiated for cocoon construction by fluorescence screening. The transcript content in the middle silk gland of the over-expressed (OE) group was twice that of the control (Fig 4A). In the RNAi group, the transcription level of *BmGlcNase1* was significantly reduced (Fig 4B). The CSW of the OE group was significantly higher than that of the control, with 10.9% increase in females (p = 0.0005) and 8.5% in males (p = 0.0038) (Fig 4A). The CSW was significantly decreased in the RNAi group, with 7% reduction in both males (p = 0.0056) and females (p = 0.0017) (Fig 4B). We compared the volume of silk glands between the transgenic strain and the control group at the beginning of the wandering stage. The results showed that compared with the control, the silk gland volume of the overexpressed strain was slightly but significantly increased (p = 0.47). Although the silk gland volume of the knock-down strain is smaller than the control group, it is not significant (0.053) (S2C and S2F Fig). Spatial expression pattern analysis showed that *BmGlcNase1* exhibited relatively high expression in the middle silk gland, while the expression level in the posterior silk gland was below the level of detection. This suggests that *BmGlcNase1* can specifically affect the synthesis of sericin. To determine whether its ectopic expression can affect fibroin synthesis, we constructed an ORF-containing vector driven by the promoter of the *fibroin heavy chain gene* and obtained the positive strain (S3 Fig). The transcription level was significantly elevated (9×) in the posterior silk gland (p<0.01) while the CSW showed no difference between the ectopic expression and the control group (S3 Fig).

These results suggest that *BmGlcNase1* can specifically affect sericin synthesis, while it does not influence fibroin synthesis. To confirm this, we surveyed the sericin content in the cocoons of the OE, RNAi, and control groups. Sericin was significantly elevated in OE cocoons, with a 13.9% and 22.5% increase in females (p<0.01) and males (p<0.01), respectively. However, the fibroin content in the two groups was similar (Fig 4C). Compared to the control, sericin in the RNAi group was significantly lower (p<0.01), with 41.2% and 27.3% reductions in females and males, respectively (Fig 4D). The fibroin content also exhibited a significant reduction (p = 0.038) in the females of the RNAi group, while the fibroin reduction in males (p = 0.067) was not significant. These data indicate that sericin is an essential component of cocoons and indispensable for normal cocoon formation. A reduction of sericin in cocoons will reduce the production of fibroin and limit silk secretion. In summary, the expression of *BmGlcNase1* affects the synthesis of sericin and cocoon formation.

### Associated variants selected during domestication and breeding

**Fig 5:**
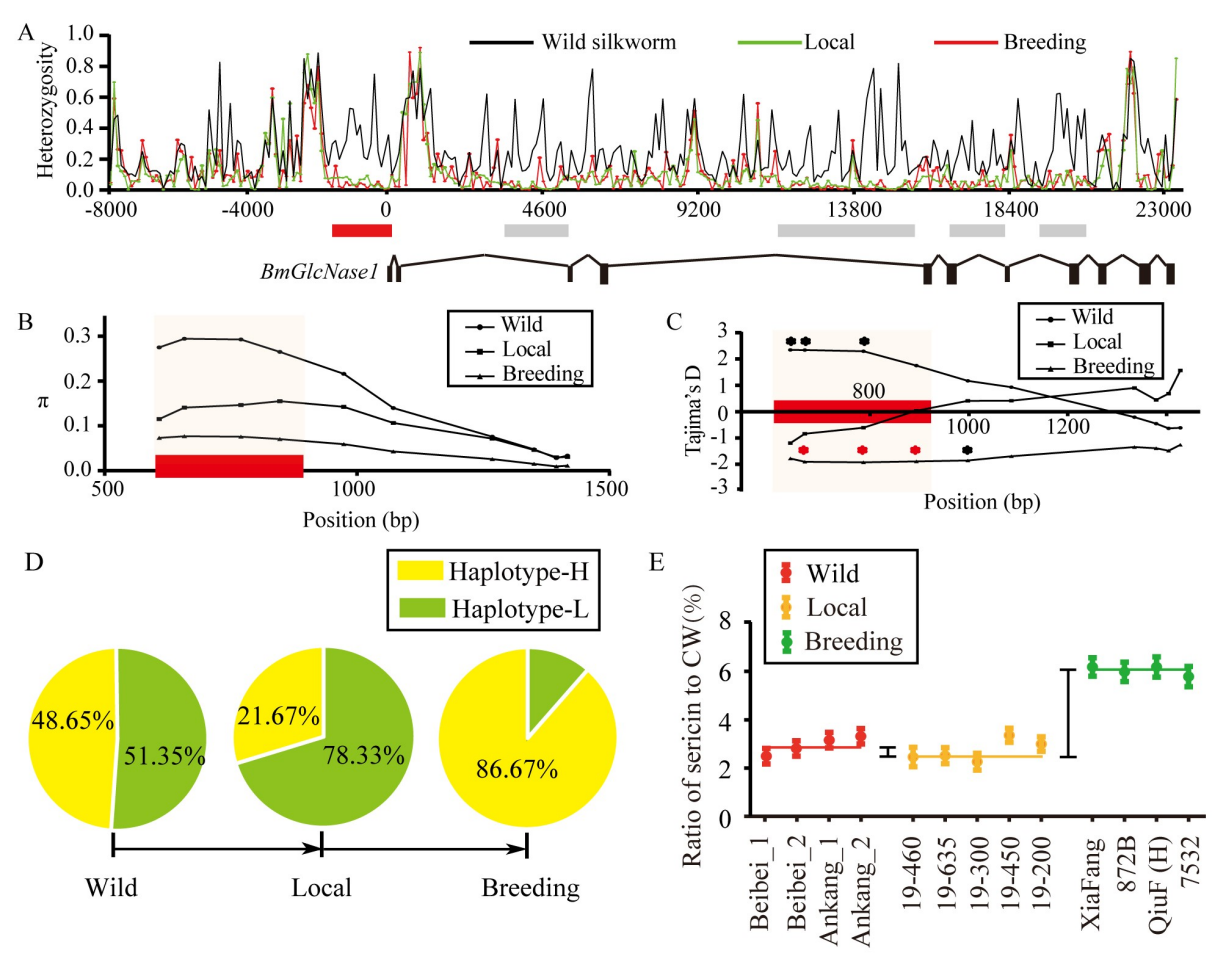
Selection analysis of *BmGlcNase1*. **(**A) Pooled heterozygosity of the genomic region of *BmGlcNase1*. The black, green and red lines represent the heterozygosity of wild silkworm (*Bombyx mandarina*), local varieties and breeding strains of Domestic silkworm (*Bombyx mori*), respectively. Numbers under the horizontal axis indicate the position relative to TSS. The thick lines below highlight the region with heterozygosity reduction in domesticated silkworms compared with the wild silkworms and the red shows the associated region upstream of *BmGlcNase1*. The wireframes indicate the structure of *BmGlcNase1*. (B-C) Sliding window analysis of nucleotide diversity (π) and of the sequenced region in wild, local, and breeding silkworm strains. The lines with circles, squares, and triangles represent wild silkworms, local varieties, and breeding strains, respectively. The black and red asterisks indicate the significance level that Tajima’D deviates from 0 is lower than 0.05 and 0.01, respectively. (D) Frequency of the haplotypes at the associated region in wild, local, and breeding strains. The yellow and green region indicate the frequency of H and L haplotype. (E) Ratio of sericin to CW in wild, local, and breeding strains. Red, orange, and green represent the ratio of sericin to cocoon weight of wild silkworm, local and breeding silkworm strains. For wild silkworm, six individuals, including three males and three females, were surveyed. For domesticated silkworms, 12 individuals, including 6 males and 6 females were surveyed. Error bar is one SD.

CSW is the most important index for silk yield and the main selection target during silkworm breeding. Genes or regions associated with CSW may also be targets of selection. Therefore, we studied the selection signals in the genomic region covering *BmGlcNase1*, and we focused on associated regions. For this study, 20 wild silkworms (*Bombyx mandarina*), 30 local varieties, and 30 breeding strains were collected (S8 Table) to detect the selection signals from domestication and breeding. Using long fragment amplification, we determined the genomic sequences covering *BmGlcNase1* from these strains. We created a balanced mix of these strains to construct three DNA pools for NGS. We obtained 1.85, 1.89, and 2.31 -giga base pair (Gb) of clean sequencing data for the wild, local, and breeding pools respectively, which resulted in a depth of more than 60,000×. Based on this, the pooled heterozygosity (Hp) was calculated. Hp in the wild silkworm pool was significantly higher than in the other two silkworm pools, with an average of 0.256 for wild strains. Hp was 0.113 and 0.120 for the local varieties and breeding strains, respectively (Fig 5A). This indicates that strong selection occurred in this region during domestication. Five regions, including the associated region upstream of TSS, were identified with greatly reduced heterozygosity. Hp of this region was 0.056 and 0.043 in the local variety pool and breeding pool, respectively. These values are much lower than values for the total region, suggesting strong selection. The Hp of the flanking region was much higher than that of the associated site in all three of the pools. This suggests that the reduction of Hp in the local and breeding pools is not the result of a hitchhiking effect.

We sequenced the associated region in wild and domesticated silkworm strain via PCR amplification. We obtained the sequence of a 750 bp region around the associated site in 12 wild, 22 local, and 26 breeding strains (S1 File). For the entire sequence region, the nucleotide diversity (*π*) was 0.16419 in the wild silkworm strains, 0.09161 in local strains, and 0.04462 in breeding strains. This illustrates a gradual reduction of nucleotide diversity from wild to local and from local to breeding silkworm strains (Table 1). The sliding window analysis of the whole sequence suggests that the biggest difference among the three populations is within the associated region (Fig 5B). A neutral test based on sliding window analysis showed that Tajima’s *D* and Fu and Li’s *F* values are both lower than 0 in domesticated silkworm strains, and values were particularly low in breeding strains (Fig 5C and Table 1). These results indicate that the associated region was subject to strong selection in domestication, especially in the breeding strains.

**Table 1:** Nucleotide diversity and neutral tests.

Test sets	S	*π*	*θ*	Tajima’s D	FL F
**Wild**	106	0.16419	0.12288	1.57098	1.68138
**Domestic**	104	0.06859	0.08264	-0.61017	0.55254
**Local**	104	0.09161	0.10061	-0.36179	0.72554
**Breeding**	95	0.04462	0.08464	-1.85127*	-0.58015

*π*, nucleotide diversity per site

*θ*, Watterson’s estimator of 4*Neμ*

FL F, Fu and Li’s F test

S, number of segregating sites.

* Significant at a probability level of 0.05.

The local varieties and breeding strains exhibited opposite haplotype patterns for the associated region (S5 Table). The ratio of haplotype-L in 30 local varieties was 78.33%, while the ratio of haplotype-H was 86.67% in the breeding populations (Fig 5D). This suggests that the two haplotypes were successively purified during domestication and breeding. The association between *BmGlcNase1* and sericin synthesis suggests that sericin synthesis was reduced during silkworm domestication but elevated during the improvement stage. To test this inference, we studied the ratio of sericin to cocoon weight (summation weight of cocoon shell and pupae, CW) of several wild silkworms, local varieties, and breeds. Compared to the wild silkworm, the relative sericin content in local varieties was slightly decreased (3.13% vs. 3.24%). However, this ratio was significantly elevated in breeding strains during the improvement stage compared to local strains (6.67% vs. 3.13%, p<0.01) (Fig 5E). These results suggest that the associated region upstream of *BmGlcNase1* was subjected to selection during domestication and breeding. In the domestication process, haplotype-L, which is related to lower CSW, was purified. This resulted in lower sericin synthesis in local varieties. In the breeding process, haplotype-H, related to higher CSW, was selected. This led to higher sericin content in breeding silkworm strains.

### *GlcNase1* was differentially selected between insects with, and without cocoons

We demonstrated that *BmGlcNase1* influences silkworm cocoons by affecting sericin synthesis. Orthologues of *BmGlcNase1* are also expressed in the silk gland of wild silkworms and other cocoon spinning insects of Bombycoidea [16–17]. To determine the relationships between *GlcNase1* and cocoon formation in other insects, we studied the presence of differential selection on *GlcNase1* in insects with and without cocoons by estimating the ratio (*ω*) of non-synonymous to synonymous substitution rates using a likelihood method. We selected 3 Lepidoptera and 3 Hymenoptera species that form cocoons and six species, lacking cocoons, that also belong to Lepidoptera and Hymenoptera (S6 Fig). Assuming that the *GlcNase1* sequences from all of the branches in the tree of the 12 taxa have the same *ω* (model A in Table 2), we estimated that *ω* = 0.06912. Next, we tested whether a model that allows different *ω* values for insects, with and without cocoons (model B in Table 2), fits the data significantly better than model A. In model B, the *ω* for insects with cocoons was 0.04843, while the *ω* for insects lacking cocoons was 0.15417. Model A and model B were significantly different (p value = 0.0398), suggesting that the selection pressure on *GlcNase1* is different between the selected insects with cocoons and those lacking a cocoon. These results suggest that *GlcNase1* might have been subject to strong purifying selection during the evolution of the cocoon trait.

**Table 2:** Likelihood ratio tests of selective pressures on *Glcnase1* in insects.

Models	*ω* (dN/dS)	lnL^a^	np^b^	Models Compared	p values
A. All of the branches have one ω0	ω0 = 0.06543	-17327.53	31	A vs. B	0.0369
B. Ancestral branches of cocooning insects have ω1, Ancestral branches of non-cocooning insects have ω2	ω1 = 0.04427; ω2 = 0.15417;	-17326.25	33		

^a^. Natural logarithm of the likelihood value.

^b^. Number of parameters.

## Discussion

### Potential roles of *BmGlcNase1* and hexosamine flux in silk protein synthesis

*BmGlcNase1* encodes the *β-1*,*4-N-acetylglucosaminidase 1* protein, which is responsible for hydrolyzing N-linked hexosamines on some proteins. Hexosamino-glycosylation is a type of protein with post-translational modification. Hexosamino-glycosylation includes N-linked and O-linked types and both types are key steps of the hexosamine signaling pathway [18]. This pathway is assumed to be a nutrient sensor [19]; the concentrations of UDP-GlcNAc are sensitive to glucose titer. UDP-GlcNAc content could influence the hexosamine modification of proteins and then regulate cell proliferation [20, 21] and oncogenesis [22, 23]. Intracellular hexosamine flux can affect the N-glycan number and the degree of branching in surface glycoprotein via cooperation with the Golgi pathway [24]. Interestingly, the receptors promoting growth, such as the EGF receptor (EGFR), harbor more N-glycan sites, while the receptors inhibiting growth possess fewer N-glycan sites. The former shows a hyperbolic response when intracellular UDP-GlcNAc content increases, while the latter exhibits a switch-like response. Thus, elevated intracellular UDP-GlcNAc content will first activate the growth-promoting receptors and facilitate the growth of cells and organs. *BmGlcNase1* has no transmembrane structure, which indicates that it may be a nucleocytoplasmic protein that hydrolyzes GlcNAc from nucleocytoplasmic glycoproteins. This may elevate the hexosamine flux, which would activate growth-promoting receptors to facilitate the development of the silk glands. However, N-hexosamine studies in insects have focused on chitin metabolism. For example, genes in the *acetylglucosaminidase* family have been reported to degrade chitin and were therefore termed *chitinases*. We genome widely identified the *acetylglucosaminidase* genes in silkworm. In addition to *BmGlcNase1*, the results revealed nine additional genes that were annotated as *acetylglucosaminidase*. These genes are expressed in various tissues where chitin is absent, such as fat body, testes, ovaries, and hemolymph (S4 Fig). This suggests that they have other functions besides chitin metabolism. Among them, *BmGlcNase1* was highly expressed in the middle silk glands. Considering the functions of N-glycan related to cell proliferation, oncogenesis, and protein synthesis in mammals, *BmGlcNase1* and hexosamine flux might also be involved in regulating the proliferation of silk gland cells, the development of the middle silk gland, and sericin protein synthesis.

### Selection pattern of *BmGlcNase1* and its effect on sericin content in cocoons

Selection analysis showed that haplotype-L and haplotype-H were purified during domestication and breeding, respectively. Domestication resulted in a decrease in the ratio of sericin to CW, and breeding resulted in an increase in this ratio. During the domestication and breeding process, easy degumming and high reel-ability (% of silk that can be reeled) of cocoons were breeder targets. The sericin content of cocoons affects the silk reeling index. Excessive sericin in cocoons will increase energy consumption and decrease the reel-ability because of the increased adhesion among the silk fibers [25]. Thus, selection has led to a reduction of sericin in the cocoon. We calculated the ratio of sericin in the cocoon shell of wild, local, and breeding strains. The ratio was much lower in local varieties than in wild silkworms (33.7–26.8% decrease), but the reduction was somewhat less when comparing local strains to breeding strains (26.8–23.9%) (S5 Fig). Early domestication may have rapidly reduced the ratio of sericin to the cocoon shell. When this ratio was reduced to a certain level, breeding selection was unable to further decrease the sericin content. During the latter process, the cocoon shell ratio (CSR) (ratio of cocoon shell weight to cocoon weight), greatly increased (0.3–0.1). This led to increased silk protein synthesis efficiency, including sericin. These data may explain why breeding silkworm strains have a higher sericin synthesis efficiency than local strains. They also indicate that a certain amount of sericin is necessary for silk fiber spinning and cocoon construction.

### Contribution of sericin to silkworm cocoon construction

Functional validation of *BmGlcNase1* demonstrated that increases in sericin have no effect on fibroin synthesis, while decreases in sericin may slightly reduce the content of fibroin in cocoons. This shows that sericin is an essential component of silkworm cocoons and the fibrosis of silk proteins is a critical process for cocoon spinning. This process is accompanied by the dehydration of fibroin and the exchange of ions. Sericin plays a critical role in this process. It absorbs water lost during the dehydration of fibroin and it also mediates the ion exchange during the fibrosis of fibroin. Sericin promotes exocrine processes by avoiding premature fibrosis of silk fibroin and protecting the wall of spinnerets [26]. When wild silkworm cocoons are spun, sericin-coated silk fibers are used to wrap a layer of mulberry leaves around the cocoon for camouflage. Thus, sericin is needed for the fibrosis of silk fiber and, the formation and concealment of silkworm cocoons.

### The evolution of *Glcnase1* and cocoon construction in insects

Insects that form cocoons occur in many evolutionarily distant orders suggesting that this trait may be the result of convergent evolution. The selective pressure on *GlcNase1* in insects with cocoons and insects without cocoons would be significantly different, suggesting that *GlcNase1* may be involved in the cocoon formation of many insect species. The *ω* (dN/dS) for both types of insects is less than 0.25. This indicates that it was subjected to purifying selection in all of the surveyed insects. Homologs of *BmGlcNase1* have multiple functions. Many members of this gene family are involved in chitin hydrolysis [27, 28]. Chitin is the main component of the exoskeleton, and the coding sequence conservation of *GlcNase1* indicates that it may be involved in chitin metabolism in insects not forming cocoons. *GlcNase1* may have evolved new functions related to cocoon formation in insects with cocoons. In addition, the phylogeny constructed by the coding sequence of *GlcNase1* showed similarity to the surveyed species, rather than a clustered distribution based on species with, or without, cocoons (S7 Fig). This suggests that *GlcNase1* independently evolved cocoon-related functions in different insects. Convergent evolution can emerge in two ways: parallel genetic evolution and collateral genetic evolution. Parallel genetic evolution refers to mutations that arise and spread in independent lineages, and collateral genetic evolution refers to mutations that either arise from a common ancestor or result from hybridization [29]. The phylogeny of the focal genes will generally be congruent with that of the species in parallel evolution [30, 31], while the phylogeny of the focal genes will be inconsistent with the phylogenetic history of species in collateral evolution [32–34]. Thus, the phylogeny of *GlcNase1* and its role in insect cocooning may be an example of parallel genetic evolution.

We investigated whether the potential neofunctionalization of *GlcNase1* results from convergent/parallel sites. Sequence alignment showed that the Glycohydro_20b2 domain at the N’ end was poorly conserved and no specific conserved amino acid sequence was detected. The Glycohydro_20 domain at the C’ end shows a higher degree of conservation in all insects (S8 Fig). The amino acids at 215, 255, 484, and 609 sites were highly conserved in insects with cocoons (S7 Fig), while they showed a high diversity in insects without cocoons. This was especially true for the amino acid variation at sites 255 and 484 located in the Glycohydro_20 domain. This suggests that the conservative amino acid sites in insects with cocoons may be involved in the neofunctionalization of *GlcNase1*.

### Conclusion

We demonstrated that the gene *BmGlcNase1* affects the synthesis of sericin. The gene was selected by domestication and breeding. Selection significantly reduced the sericin content in domestic silkworm cocoons compared to the content in its wild ancestor. These data, combined with the functional conservation of *GlcNase1* in insects with cocoons, suggest that *BmGlcNase1* is involved in the cocoon process of silkworms and in other insects with cocoons.

## Materials and Methods

### Silkworm samples

We used two populations of silkworms for genetic analysis. An extreme phenotype population for selective genotyping and germlines for association analysis. To construct the former population, two silkworm strains (IS-Dazao and 872B), which were used in preliminary QTL analysis, were selected as parents to produce a backcross population, IS-Dazao♀×(IS-Dazao×872B)F_1_♂. The individuals in this population were reared on fresh mulberry leaves to the cocoon stage at 25°C with a 12:12 h (L:D) photoperiod. At the eye coloring stage, the cocoons of each individual were opened to distinguish the sexes based on features of the pupal abdomen, and the cocoon shell weight (CSW) was measured for the two sexes. Based on the phenotype data, male individuals with extreme CSW values were selected from the progeny of a single female moth. The number of individuals with extremely high and extremely low CSW (H sub-population and L sub-population, respectively) made up approximately 10% of the total males in the mapping population. The two sub-populations were used as the extreme phenotype populations for selective genotyping. For association analysis, 95 germlines were chosen from the silkworm gene bank of Southwest University (Chongqing, China) with three selection criteria: the strains had normal viability, geographical or systematical source silkworm strains should be as diverse as possible to avoid the possible kinship and they should exhibited substantial CSW variation (S8 Table). These silkworm strains were reared on fresh mulberry leaves at 25°C with a 12:12 h (L:D) photoperiod. At the pupal stage, 100 males and 100 females were selected from each germline for CSW determination using the method detailed above. Genomic DNA of individuals from the two populations was obtained via a standard phenol-chloroform extraction method [35].

### Genotyping and statistics for genetic analysis

To narrow the mapping region by selective genotyping, we developed 18 indel markers in the preliminary mapping region (S1 Table). These were genotyped via PCR amplification and polyacrylamide gel electrophoresis (PAGE). The linkage significance between these indels and CSW was calculated by one-way ANOVA. After genotyping analysis, the mapping region was narrowed to a 270 kb region. We then developed 99 indel markers in this region for association analysis. These were also genotyped by PCR amplification and PAGE. The association significance between indels and CSW was calculated using one-way ANOVA. Primers used to amplify these indels are listed in S2 Table.

### Sampling and expression pattern analysis

To investigate the expression level of *BmGlcNase1*, various silkworm tissues were dissected from IS-Dazao larvae on the third day of the 5^th^ instar and placed into cold normal saline (NS). The dissected tissues included muscle, testis, wing disc, midgut, malpighian tubules, anterior-middle silk gland, fat body, ovary, head, posterior silk gland, integument, and hemolymph. For temporal expression pattern analysis, 17 samples were selected beginning at 3 h after incubation and ending at day seven of the 5^th^ instar. At the embryonic stage, eggs laid by one female moth were divided into three groups and preserved in three tubes with TRIzol (Invitrogen, USA). From the first day of the first instar to the molting stage of the 4^th^ instar, whole larvae were preserved. At the 5^th^ instar, the anterior-middle silk glands were dissected from larvae each day. For the association analysis between expression and CSW variation, 36 germlines (S8 Table) with varied CSW values were selected and reared to the final day of the fifth instar. Middle silk glands were dissected on this day from each germline. All of these samples were preserved in cold TRIzol. Total RNA was extracted and purified using TRIzol (Invitrogen, USA) according to the manufacturer instructions. Then, the first strand cDNA was generated via reverse transcription using a PrimeScript RT Reagent Kit (Takara, Japan) according to the supplier instructions. The correlation between the expression level of *BmGlcNase1* in the silk glands of each strain and CSW was analyzed by linear regression. Analysis of the difference in expression of *BmGlcNase1* between different groups was conducted using Student’s *t*-test. For each strain, three replicates were performed and each replicate contained middle silk glands from three larvae. Primers used for real-time PCR and RT-PCR are listed in S6 Table.

### Amplification and sequencing

To study the sequence variation between parents for the full-length cDNA of *BmGlcNase1*, we performed cDNA cloning and rapid amplification of cDNA ends (RACE). The total RNA was extracted from the middle silk gland of parents on the final day of the 5^th^ instar according to the procedures detailed above. For cDNA cloning, four pairs of primers were designed (S3A Table). Two strands of primer for double-end RACE amplification were also designed based on the inner cDNA sequence. The RACE amplification was performed using the instructions of the Marathon cDNA Amplification Kit (Takara). All of these products were then inserted into a pM19-T vector (Takara, Japan) and sequenced. To obtain the genomic sequence of *BmGlcNase1*, we designed 21 pairs of primers to successively amplify the 30 kb genomic region. These products were also inserted into a pM19-T vector (Takara, Japan) and sequenced. All of the primers used for sequencing were listed in S3B Table. Primers for association analysis of the Indels in upstream region of *BmGlcNase1* were listed in S4 Table.

### Transgenic function analysis

Transgenic-based over-expression and knock-down were used to validate the function of *BmGlcNase1*. The *Ser1* and *FibH* promoters used to drive the expression of *BmGlcNase1* in middle and posterior silk glands were synthesized based on the sequences in Wang et al. [36] and Zhao et al. [37]. The primers, ORF/RNAi fragment, and SV40 termination sequence were then sequentially linked together with the restriction sites *NotI* and *kpnI*. The expression core was then inserted into the PiggyBac transposon with dsRed and EGFP, both of which were driven by 3 × p3 promoter. Primers used for vector construction were list in S7 Table. The over-expression and knock-down constructs, accompanied with a helper vector, were then, respectively, injected into the newly laid non-diapause eggs of IS-Dazao (incubated at 16°C) and 305, a multivoltine silkworm strain with a relatively high CSW. The new G1 larvae were then subjected to fluorescence screening to identify the positive G1 individuals. Through back-crosses and self-crosses with control lines, we established seven overexpression, two knock down and two posterior silk gland ectopic expression lines. By detecting the expression level of the *BmGlcNase1* gene of each line, we selected the line with the highest overexpression and interference effects for the following experiment. Inverse PCR was then performed to identify the insertion sites of transgenic silkworms according to the method of Wang et al. [36]. To accurately evaluate the CSW of transgenic strains and the corresponding control, we reared 150 positive and 150 control individuals together to remove interference from environmental differences. On the last day of the 5^th^ instar, fluorescence screening was used to distinguish the positive individuals from controls. The CSW values of these individuals were then measured using the method detailed above. The fibroin content of cocoons of various strains was determined by degumming based on the sodium carbonate solution reported by Dai et al. [38]. Cocoons were heat-dried at 70°C for 7 h and weighted. Then the dried cocoons were boiled in 150 mL 0.5% sodium–carbonate solution at 100°C for 2 h, followed by washing 45 min with deionized water. Degummed cocoons were dried at 65°C for 24 h and weighted. The corresponding sericin content was calculated by the following formula: Weight_(Sericin)_ = CSW-Weight_(Fibrion)_.

### Selection analysis in domestication and breeding

A total of 20 wild (*B*. *mandarina*), 30 local, and 30 breeding silkworm strains (S8 Table) were selected for pooled heterozygosity investigation. To capture the genomic fragment in these strains, four pairs of primers with an average product length of 8327 bp were designed. These were amplified via LA-Taq (Takara, Japan) under the conditions specified in the instructions. This product was purified by the Gel Extraction Kit (Omega, USA) using manufacturer instructions. Then, the products were equally mixed to produce the wild, local, and breeding pools. The Illumina sequencing libraries were constructed and then subjected to sequencing under the paired-end mode on Illumina HiSeq 2500 (Novogene, Beijing, China). We generated approximately 1.85, 1.89, and 2.31 Gb raw reads for wild, local, and breeding pool, respectively. The genomic sequence of *BmGlcNase1* in IS-Dazao obtained in this research was used as a reference. The raw data were then subjected to joint trimming, low-quality data filtering, comparison with the reference genome, and variant calling. Based on this, the Hp of each sample was calculated based on the method of Erik [39]. Multi-sequence alignment of nucleic acid sequences was conducted by using mega software (muscle algorithm). Through DNAsp v6 software [40], the nucleotide polymorphism (pi), Tajima ‘s D value and Fu and Li ‘s F value were calculated by sliding window (window size: 100 bp, sliding step size: 25 bp). Sericin content in the cocoons of wild, local, and breeding strains was also determined using the method above. The weight ratios of sericin to cocoon and to cocoon shell were calculated using the following equations:

Ratio(sericin/cocoon)=weightofsericin/weightoftotalcocoon;

Ratio(sericin/cocoonshell)=weightofsericin/CSW.

### Selection pressure analysis

To estimate selection pressures on the *GlcNase1* gene in cocooning insects and non-cocooning insects, we selected six cocooning insect species and six non-cocooning insect species (S9A Fig). The *GlcNase1* of *Bombyx mandarina* was from Silkbase (http://silkbase.ab.a.u-tokyo.ac.jp/cgi-bin/index.cgi). Transcript sequence of *Antheraea pernyi GlcNase1* was from the transcriptome Shotgun Assemblies in Dong *et al*. [17]. Genome and proteome data of the other species were downloaded from NCBI by the accessions as follows: GCA_000572035.2 (*Microplitis demolitor*), GCA_000806365.1 (*Fopius arisanus*), GCA_001412515.3 (*Diachasma alloeum*), GCA_002156985.1 (*Helicoverpa armigera*), GCA_000836235.1 (*Papilio xuthus*), GCA_009193385.2 (*Nasonia vitripennis*), GCA_000599845.3 (*Trichogramma pretiosum*), GCA_001465965.1 (*Polistes dominula*) and GCA_001266575.1 (*Operophtera brumata*). The protein sequence in each species was searched by pblast, and then the corresponding transcript sequence was obtained by tblastn. Each transcript was mapped to the corresponding genome by blastn to obtain its genomic location information and determine the copy number of *GlcNase1* in each species. Real-time PCR was used to compare the DNA content of the *Antheraea pernyi GlcNase1* relative to the housekeeping genes, *eukaryotic translation initiation factor 4A* (*ETI-4*) and *Actin3* (S6 Table), to determine the genome copy number (S9B Fig). After confirmation of the *GlcNase1* copy number of each species, the transcript sequences were aligned using ClustalW (codon) followed by manual adjustments (S6 Fig). A maximum-likelihood method was used to estimate the selective pressures using branch models in codeml of the EasyCodeML package [41]. The ratio of non-synonymous to synonymous substitution rates, termed ω, was used to estimate the mean selection pressures on different branches of the tree. We first estimated ω across the tree under a one-ratio model. Then, we estimated an independent ω value for cocoon forming and non-cocoon forming branches under the free-ratio model. The maximum-likelihood tree of *GlcNase1* was constructed by MEGA7.1.0 [42] with 1000 bootstrap runs.

## Supporting information

S1 Fig Expression pattern of *BmGlcNase1* in various tissues at the third day of the 5^th^ instar larva. (A) Expression level of *BmGlcNase1* in various tissues. M_tube stands for malpighian tube; MSG and PSG indicate the middle and the posterior silk gland respectively. Three replicates were performed. Error bar, SD. (B) Silk gland volume of IS-Dazao and 872B. V-silk gland indicates the volume of silk gland. ** represents a significant difference at the 0.01 level.
(PDF)

S2 Fig Screening of transgenic positive lines and detection of insertion sites. (A, D) The positive line over-expressing and knock-down *BmGlcNase1*. The above sentences show the expression core of the corresponding construct. White, EGFP, and Red indicate the positive individual under white, green, and red light irradiation. From top to bottom, the pictures show the luminescence of the compound eyes of embryos and adults. The scales in the pictures of new incubated larva and adult are 1 mm and 1 cm respectively. (B, E) The insertion site of over-expression and transgenic knock-down construct. The above shows the structure of the transgenic construct. Arrows, red, green boxes and white boxes indicate promoters, Red fluorescent protein, Enhanced green fluorescent protein, and other elements in the vectors. The bottom shows the insertion sites of the constructs. (C, F) Comparison of the silk gland volume between control and transgenic silkworm. OE and RNAi, over-expression and knock down individuals. N = 12.
(PDF)

S3 Fig Ectopic expression of *BmGlcNase1* in the posterior silk gland. (A) Screening of positive ectopic expression lines. The above sentences show the expression core of the ectopic expression construct. White, EGFP, and Red indicate the positive individuals under white, green and red light irradiation. From top to bottom, the pictures show the luminescence of the compound eyes of embryos and adults. The scales in the pictures of new incubated larva and adult are 1 mm and 1 cm respectively. (B) Insertion site of ectopic expression construct in two positive lines, Fib-1 and Fib-2. Chromosome diagrams indicate the insertion sites of the ectopic expression construct in Fib-1 and Fib-2 line. The middle represents the structure of the transgenic construct. (C–D) show the expression level of *BmGlcNase1* and the CSW, respectively, in the Fib-1 line and control. Two tailed Student’s test was used for comparison. Error bar, SD. N.S., not significant.
(PDF)

S4 Fig Expression pattern of genes of *acetylglucosaminidase* family of silkworm. M_tube stands for malpighian tube; Tr_plexus is short for Tracheal plexus; MSG and PSG indicate the middle and the posterior silk gland respectively. Actin3 gene was used as the inner control.
(PDF)

S5 Fig Ratio of sericin to cocoon shell weight in wild silkworm, local and breeding domesticated silkworm strains. The red, orange, and green represent the ratio of sericin to cocoon weight of the wild silkworm, local and breeding silkworm strains. For wild silkworm, 6 individuals, including 3 males and 3 females were studied and for domesticated silkworms, 12 individuals, including 6 males and 6 females were studied. Error bar, SD.
(PDF)

S6 Fig Nucleotide sequences of insects used for selection pressure analysis. (PDF)

S7 Fig Phylogenetic analysis of selected insects. The red and black lines highlight the insect species cocoons and without cocoon. The wireframes represent the domains in the BmGlcNase1 product. The orange triangle indicates the Glycohydro_20b2 domain and the green frame indicates the Glycohydro_20 domain. The amino acids below show the conserved sites in cocooning insects.
(PDF)

S8 Fig Protein sequence comparison of *GlcNase1* in insects with cocoons and without cocoons. The region with the black line above is the Glycohydro_20b2 domain and the region with the red line above is the Glycohydro_20 domain. Red and black lines highlight the insect species with cocoons and those without cocoons.
(PDF)

S9 Fig Information about the orthologues in other insects. (A) Information of the species, accession numbers of *GlcNase1* and the genomic location of the orthologues. (B) Copy number investigation of *GlcNase1* orthologues in *Antheraea yamamai*.
(PDF)

S1 Table Primers used for selective genotyping. (XLSX)

S2 Table Primers used for association analysis. (XLSX)

S3 Table Primers used for sequencing. (XLSX)

S4 Table Primers used for association analysis of the Indels in the upstream region of *BmGlcNase1*. (XLSX)

S5 Table Genotype of variants at the upstream of *BmGlcNase1*. (XLSX)

S6 Table Primers for real time PCR and RT-PCR. (XLSX)

S7 Table Primers used for plasmid construction. (XLSX)

S8 Table Information on the silkworm samples used. (XLSX)

S1 File Sequence information. Part 1. Full-length cDNA of *BmGlcNase1*; Part 2. Genomic sequence of *BmGlcNase1*; Part 3. Sequence for artificial selection analysis.
(TXT)
