# Supplementary figures and images for: The beta-1, 4-N-acetylglucosaminidase 1 gene, selected by domestication and breeding, is involved in cocoon construction of Bombyx mori
Source: PLoS Genet. 2020 Jul 15;16(7):e1008907. doi: 10.1371/journal.pgen.1008907 (PMC7363074; doi:10.1371/journal.pgen.1008907)

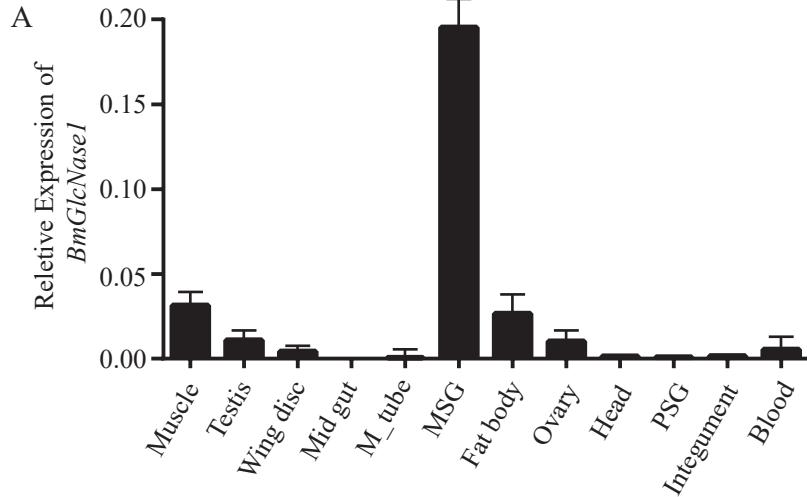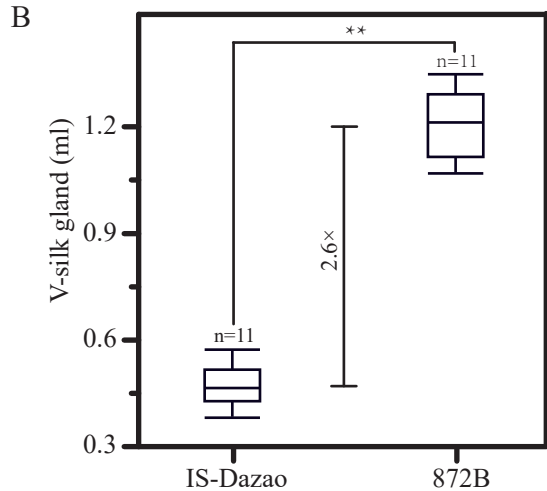

Supplement: S1 Fig — (A) Expression level of BmGlcNase1 in various tissues. M_tube stands for malpighian tube; MSG and PSG indicate the middle and the posterior silk gland respectively. Three replicates were performed. Error bar, SD. (B) Silk gland volume of IS-Dazao and 872B. V-silk gland indicates the volume of silk gland. ** represents a significant difference at the 0.01 level. (PDF) [file pgen.1008907.s001.pdf]

A

Ser1-011646-SV40

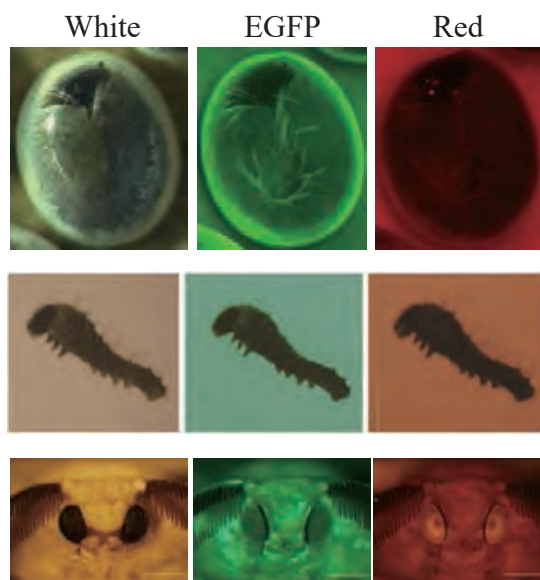

D

Ser1-011646dsRNA-SV40

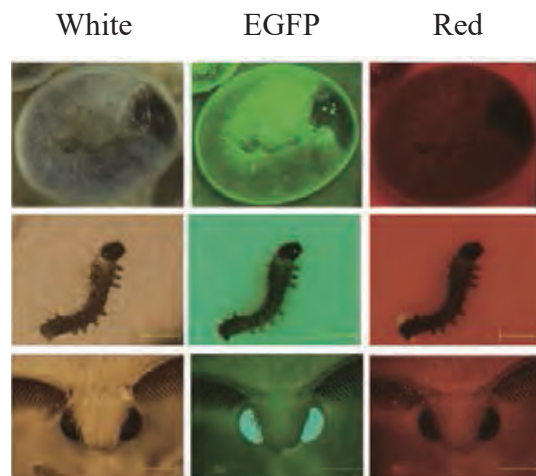

B

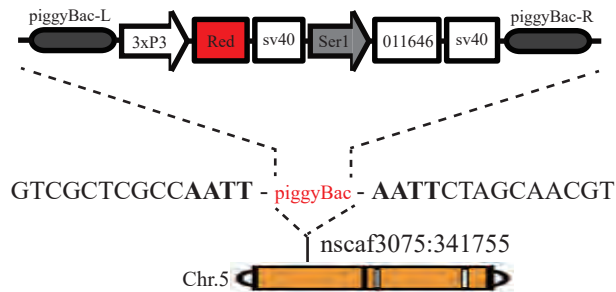

C

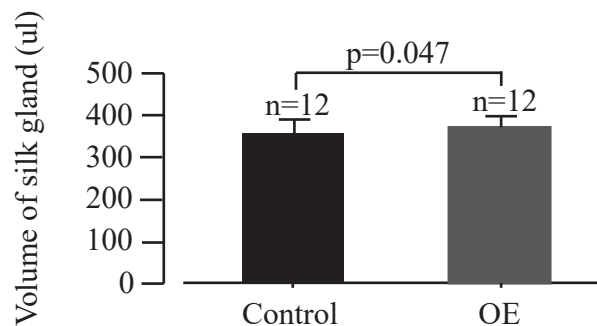

E

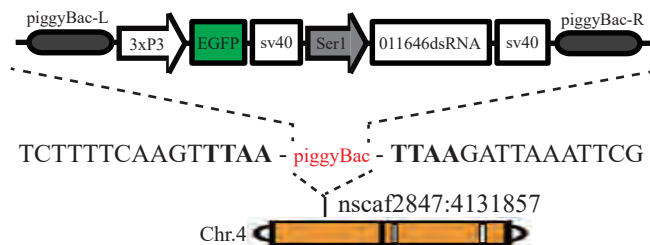

F

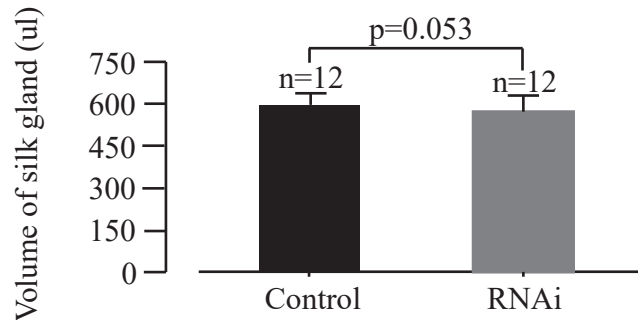

Supplement: S2 Fig — (A, D) The positive line over-expressing and knock-down BmGlcNase1. The above sentences show the expression core of the corresponding construct. White, EGFP, and Red indicate the positive individual under white, green, and red light irradiation. From top to bottom, the pictures show the luminescence of the compound eyes of embryos and adults. The scales in the pictures of new incubated larva and adult are 1 mm and 1 cm respectively. (B, E) The insertion site of over-expression and transgenic knock-down construct. The above shows the structure of the transgenic construct. Arrows, red, green boxes and white boxes indicate promoters, Red fluorescent protein, Enhanced green fluorescent protein, and other elements in the vectors. The bottom shows the insertion sites of the constructs. (C, F) Comparison of the silk gland volume between control and transgenic silkworm. OE and RNAi, over-expression and knock down individuals. N = 12. (PDF) [file pgen.1008907.s002.pdf]

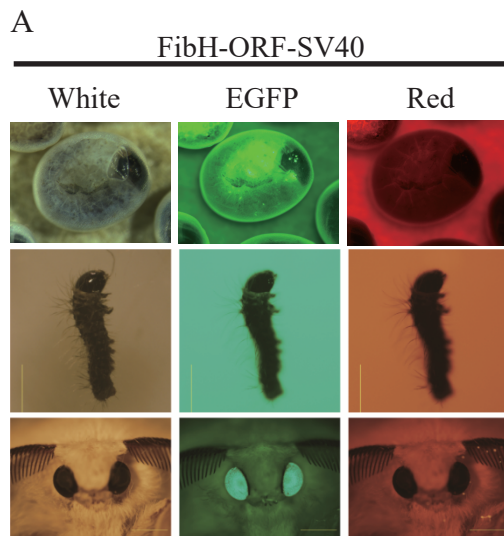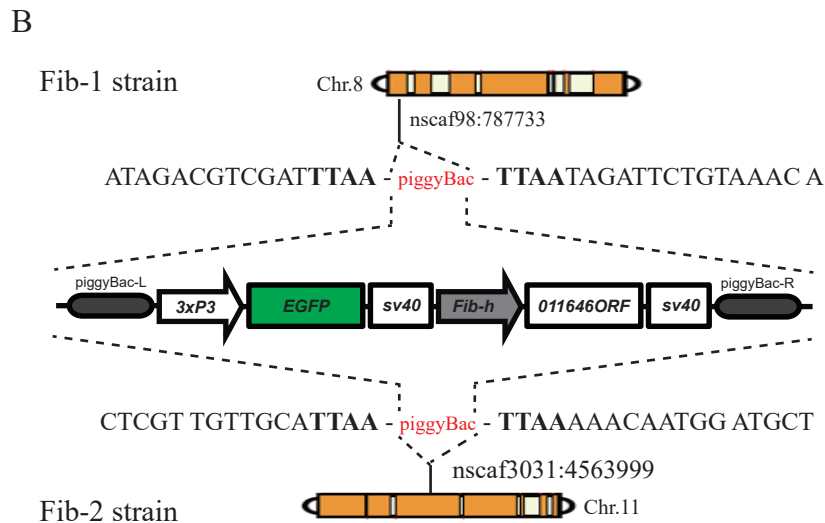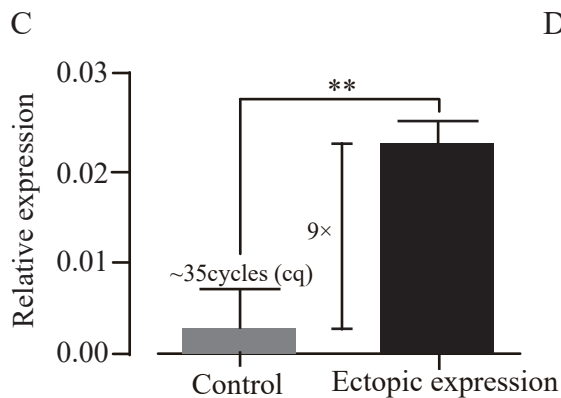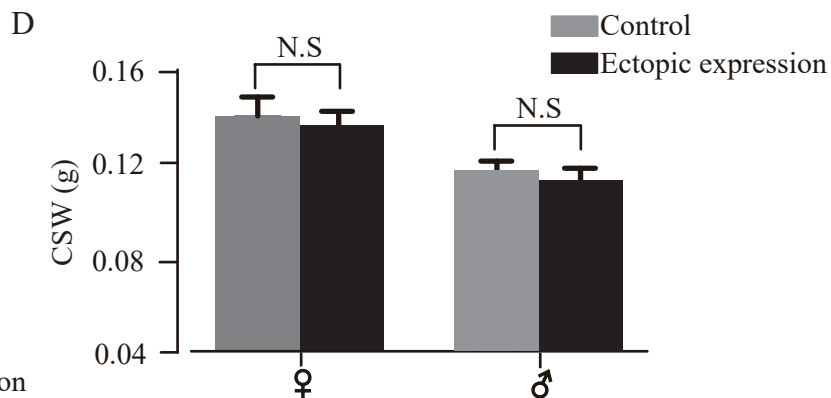

Supplement: S3 Fig — (A) Screening of positive ectopic expression lines. The above sentences show the expression core of the ectopic expression construct. White, EGFP, and Red indicate the positive individuals under white, green and red light irradiation. From top to bottom, the pictures show the luminescence of the compound eyes of embryos and adults. The scales in the pictures of new incubated larva and adult are 1 mm and 1 cm respectively. (B) Insertion site of ectopic expression construct in two positive lines, Fib-1 and Fib-2. Chromosome diagrams indicate the insertion sites of the ectopic expression construct in Fib-1 and Fib-2 line. The middle represents the structure of the transgenic construct. (C–D) show the expression level of BmGlcNase1 and the CSW, respectively, in the Fib-1 line and control. Two tailed Student’s test was used for comparison. Error bar, SD. N.S., not significant. (PDF) [file pgen.1008907.s003.pdf]

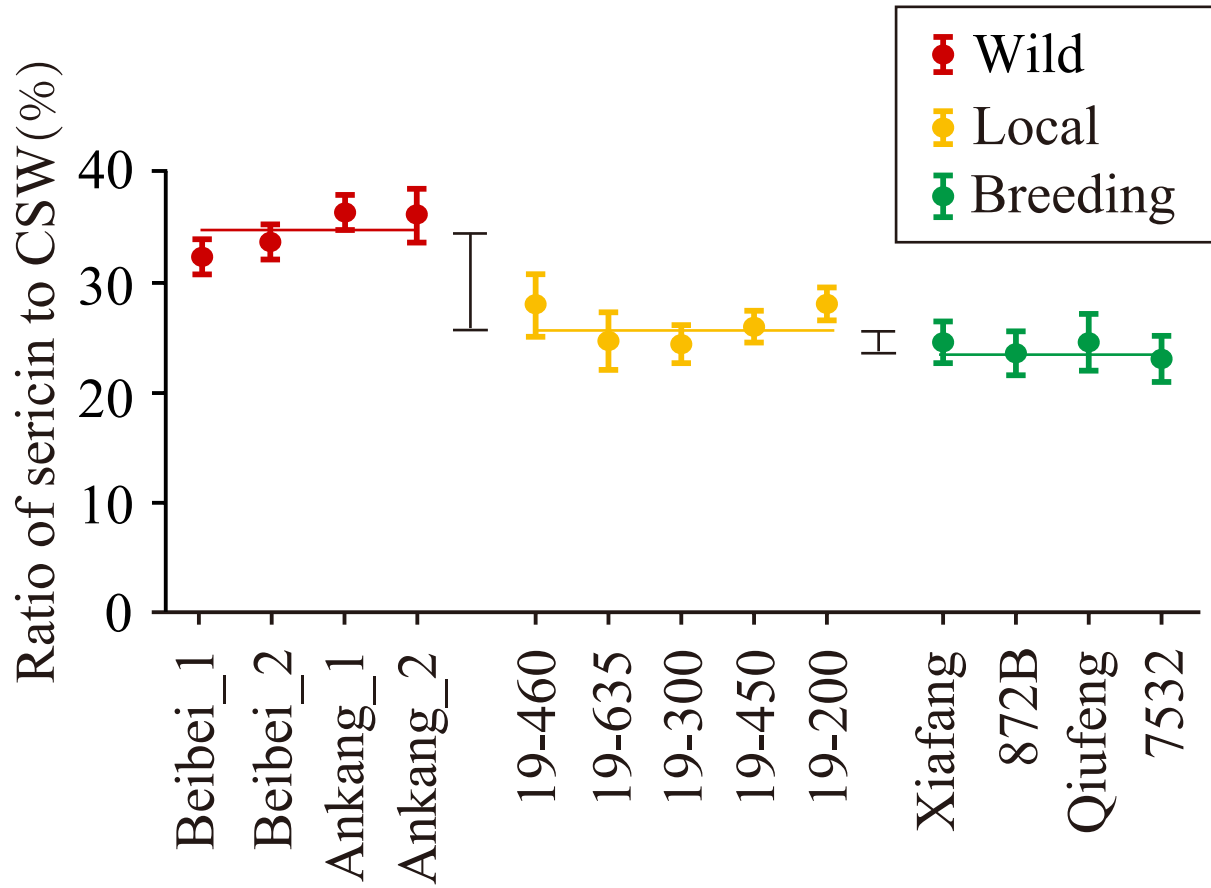

Supplement: S5 Fig — The red, orange, and green represent the ratio of sericin to cocoon weight of the wild silkworm, local and breeding silkworm strains. For wild silkworm, 6 individuals, including 3 males and 3 females were studied and for domesticated silkworms, 12 individuals, including 6 males and 6 females were studied. Error bar, SD. (PDF) [file pgen.1008907.s005.pdf]

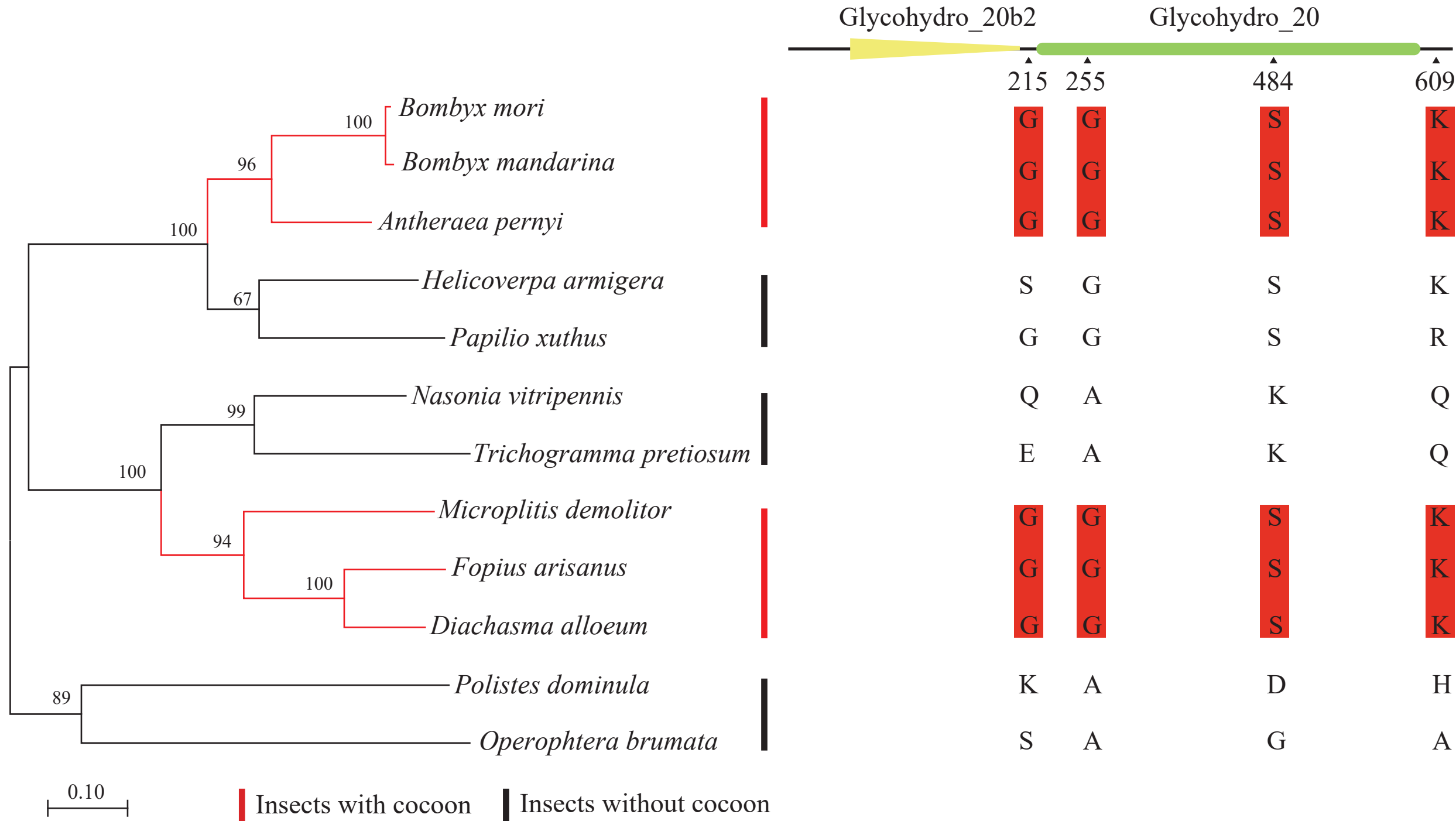

Supplement: S8 Fig — The region with the black line above is the Glycohydro_20b2 domain and the region with the red line above is the Glycohydro_20 domain. Red and black lines highlight the insect species with cocoons and those without cocoons. (PDF) [file pgen.1008907.s008.pdf]
