# Supplementary material for: The beta-1, 4-N-acetylglucosaminidase 1 gene, selected by domestication and breeding, is involved in cocoon construction of Bombyx mori
Source: PLoS Genet. 2020 Jul 15;16(7):e1008907. doi: 10.1371/journal.pgen.1008907 (PMC7363074; doi:10.1371/journal.pgen.1008907)
Supplement: S4 Fig — M_tube stands for malpighian tube; Tr_plexus is short for Tracheal plexus; MSG and PSG indicate the middle and the posterior silk gland respectively. Actin3 gene was used as the inner control. (PDF) [file pgen.1008907.s004.pdf]

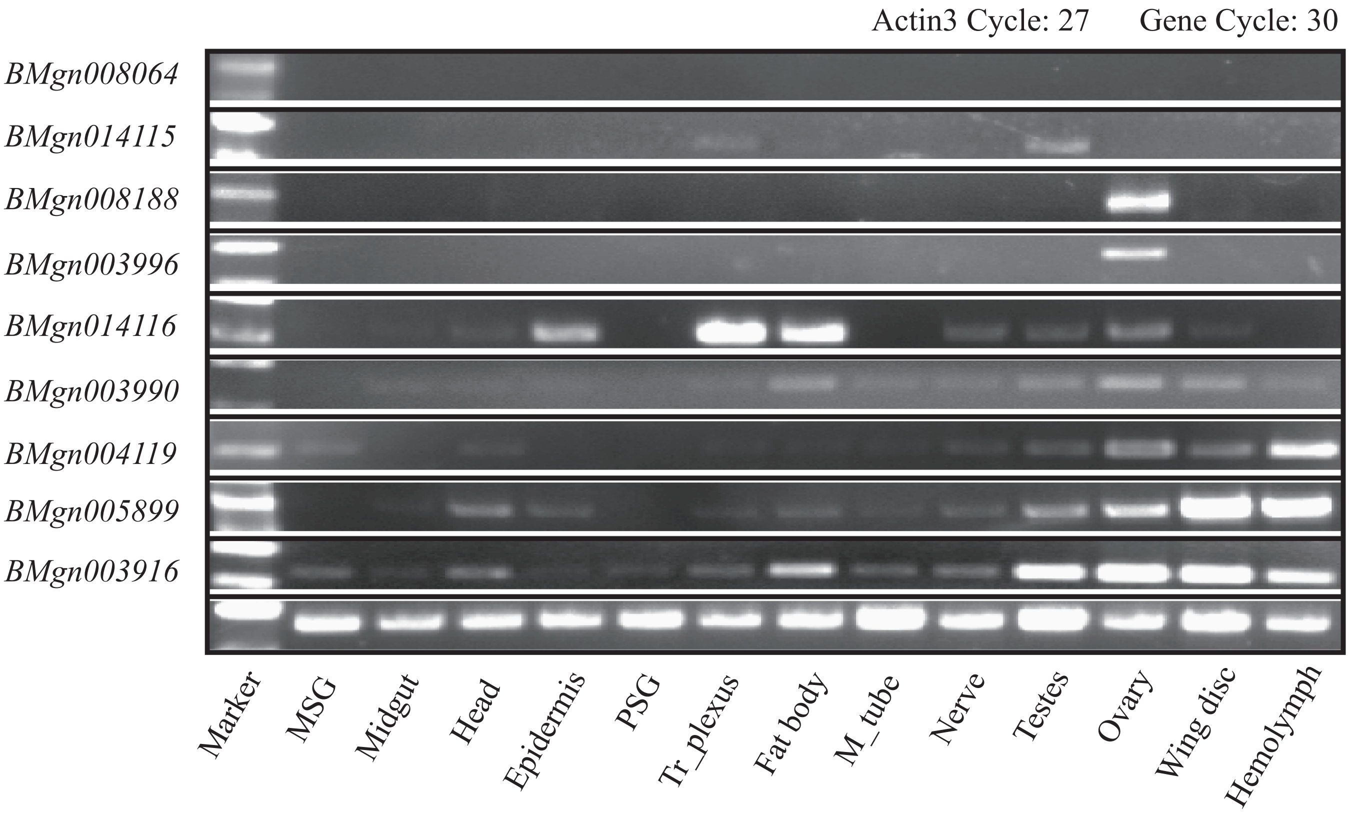

Actin3 Cycle: 27
Gene Cycle: 30
BMgn008064
BMgn014115
BMgn008188
BMgn003996
BMgn014116
BMgn003990
BMgn004119
BMgn005899
BMgn003916
Marker
MSG
Midgut
Head
Epidermis
PSG
Tr_plexus
Fat body
M_tube
Nerve
Testes
Ovary
Wing disc
Hemolymph
