# Supplementary material for: The beta-1, 4-N-acetylglucosaminidase 1 gene, selected by domestication and breeding, is involved in cocoon construction of Bombyx mori
Source: PLoS Genet. 2020 Jul 15;16(7):e1008907. doi: 10.1371/journal.pgen.1008907 (PMC7363074; doi:10.1371/journal.pgen.1008907)
Supplement: S7 Fig — The red and black lines highlight the insect species cocoons and without cocoon. The wireframes represent the domains in the BmGlcNase1 product. The orange triangle indicates the Glycohydro_20b2 domain and the green frame indicates the Glycohydro_20 domain. The amino acids below show the conserved sites in cocooning insects. (PDF) [file pgen.1008907.s007.pdf]

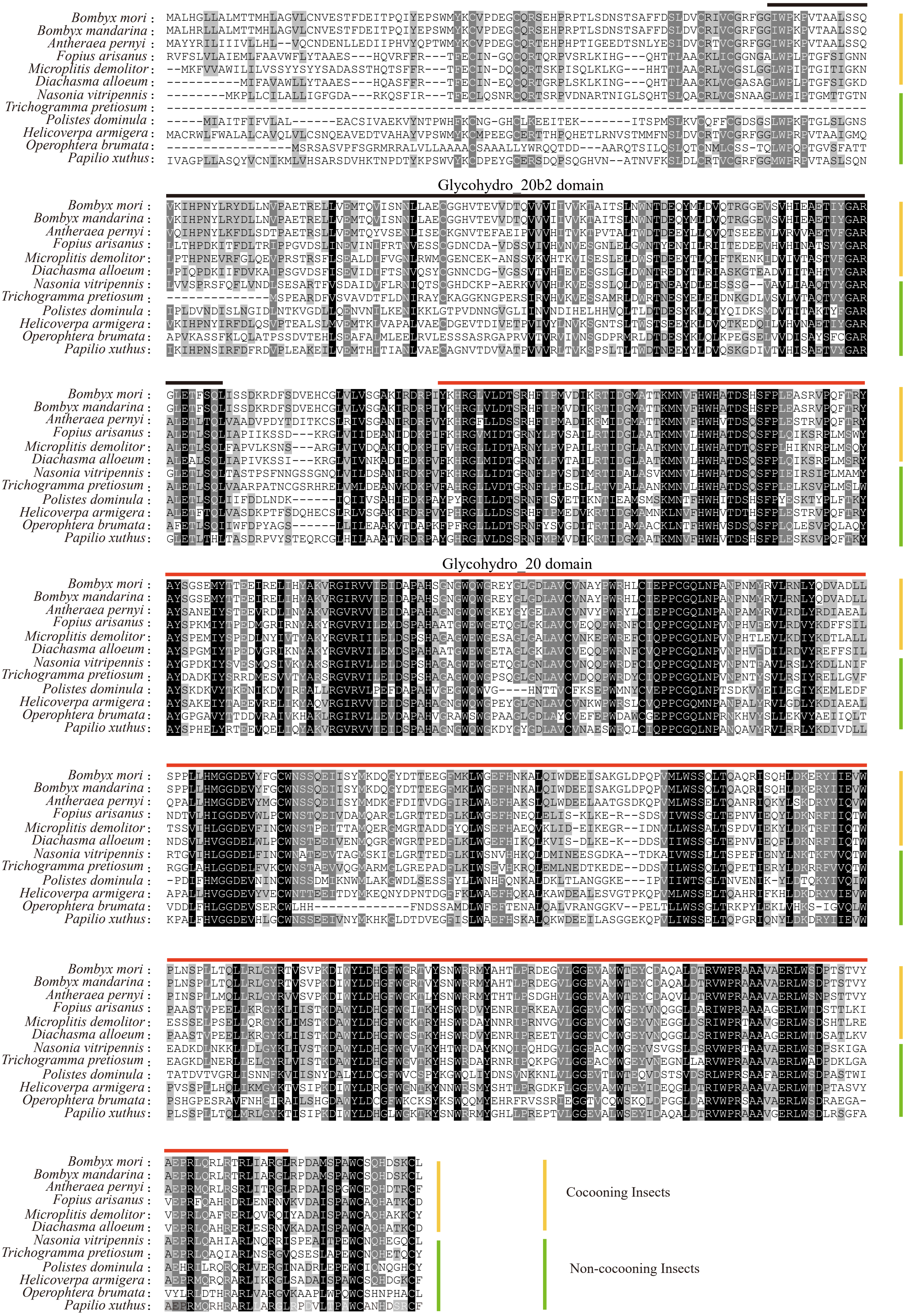
Bombyx mori :
Bombyx mandarina :
Antheraea pernyi :
Fopius arisanus :
Microplitis demolitor :
Diachasma alloeum :
Nasonia vitripennis :
Trichogramma pretiosum :
Polistes dominula :
Helicoverpa armigera :
Operophtera brumata :
Papilio xuthus :
Glycohydro_20b2 domain
Glycohydro_20 domain
Cocooning Insects
Non-cocooning Insects
