# Supplementary material for: The beta-1, 4-N-acetylglucosaminidase 1 gene, selected by domestication and breeding, is involved in cocoon construction of Bombyx mori
Source: PLoS Genet. 2020 Jul 15;16(7):e1008907. doi: 10.1371/journal.pgen.1008907 (PMC7363074; doi:10.1371/journal.pgen.1008907)

A

| Species | ID of *GlcNase1* | Genomic location |
|---|---|---|
| *Bombyx mori*<br>*Bombyx mandarina* | *BMgn011646* | chr11: 10763591-10788019 (+) |
| *Antheraea pernyi* | *GBZF01003010.1* | / |
| *Microplitis demolitor* | XP_008544080.1 | Mdem_scaffold_0120: 1910569-1913154 |
| *Fopius arisanus* | XP_011315002.1/XP_011315001.1 | ASM80636v1 scaffold000081090044 -1087244 |
| *Diachasma alloeum* | XP_015108847.1/XP_015108764.1 | Dall_scaffold_00176: 3349507-3357295 |
| *Helicoverpa armigera* | XP_021193265.1/XP_021193264.1 | Harm_1.0 scaffold_186: 108719-91965 |
| *Papilio Xuthus* | XP_013172591.1/XP_013172589.1 | Pxut_1.0 DF826945.1: 5381952-5363985 |
| *Nasonia vitripennis* | XP_001603248.2/XP_032451775.1 | CM000915.2: 386262-383685 |
| *Trichogramma pretiosum* | XP_014233011.1 | NW_014334931.1: 740606-735973 |
| *Polistes dominula* | XP_015189390.1 | unplaced genomic scaffold, Pdom r1.2: 53379 - 48713 |
| *Operophtera brumata* | KOB79240.1 | WM2013NL OBRU01_Sc07043:11671-10151 |

B

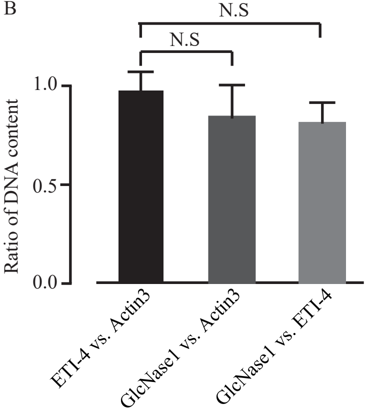

Supplement: S9 Fig — (A) Information of the species, accession numbers of GlcNase1 and the genomic location of the orthologues. (B) Copy number investigation of GlcNase1 orthologues in Antheraea yamamai. (PDF) [file pgen.1008907.s009.pdf]
